# Glucocorticoids do not influence a secondary sexual trait or its behavioral expression in eastern fence lizards

**DOI:** 10.1038/s41598-019-41596-1

**Published:** 2019-03-26

**Authors:** K. J. MacLeod, G. L. McCormick, T. Langkilde

**Affiliations:** 1 Department of Ecosystem Science and Management, The Pennsylvania State University, Forest Resources Building, University Park, PA 16802 USA; 2 Department of Biology, Intercollege Graduate Degree Program in Ecology, and Center for Brain, Behavior and Cognition, The Pennsylvania State University, University Park, PA 16802 USA

## Abstract

Secondary sexual traits and associated behaviors can be influenced by environmental factors such as exposure to stressors. Such effects may be mediated by the physiological stress response, which is typified by the release of glucocorticoid hormones. The effects of glucocorticoids on sexual traits such as plumage and display coloration have most commonly been studied in isolation rather than in conjunction with other pertinent aspects of signalling, such as behavior and habitat use, though these have substantial potential to alter signal perception. Here we test the effects of corticosterone (CORT), a common glucocorticoid, on a secondary sexual trait (badge coloration) in male eastern fence lizards (*Sceloporus undulatus*), and behaviors associated with its expression. We show that neither baseline nor experimentally manipulated CORT levels were associated with badge coloration. Further, elevation of CORT levels in the field did not alter signalling or associated territorial behaviors. There was a trend for CORT-treatment to influence perch height selection, which may influence signal perception. We suggest that future studies investigating the effects of environmental stressors and associated physiological changes on secondary sexual traits should consider behaviors and ecology relevant to signal perception in order to best understand the influence of stressors in nature.

## Introduction

Secondary sexual traits - such as bright plumage or elaborate ornamentation, and associated signalling behaviors - are important for territoriality and mate choice^1–3^. Traditional models of sexual selection have focused on the genetic basis of secondary sexual traits^4^, but such traits can also can be influenced by environmental factors, generating considerable within-individual variation in trait expression over time based on factors such as condition^5–8^. For example, exposure to environmental stressors can influence secondary sexual traits in vertebrates^9^. High population density, a common ecological stressor, is associated with reduced ventral coloration in the common lizard, *Lacerta vivipara*^10^; nutritional stress affects ornamental plumage in birds^11^; and parasite load correlates with aspects of male structural coloration in *Gallotia* lizards^12^. Given the influence of individual condition, sexual traits can function as signals of, for example, competitive and foraging ability and resistance to parasites^13–16^.

Effects of stressor exposure on sexual traits are likely to be mediated by the physiological stress response, a suite of neuroendocrine processes characterized by activation of the hypothalamic-pituitary-adrenal axis (HPA) and subsequent release of glucocorticoid hormones which facilitate appropriate reactions to and recovery from stressors^17–19^. Links between glucocorticoids and the expression of carotenoid-based coloration are well-established^20–23^, potentially due to glucocorticoid-driven redirection of carotenoids (which are primarily antioxidants) to immune functions^24^. Glucocorticoids can also influence melanic coloration^25^ through inhibiting effects on melanogenesis^26^. Although stress effects on primarily visual traits such as plumage and display coloration have most commonly been studied in isolation^27,28^, color frequently functions as part of a multi-modal display which also includes behavioral components^28–30^. Given the common effects of stress on behavior (for example, increased vigilance and shelter-seeking behaviors^9^), it seems likely that exposure to environmental stressors should also influence behavioral components of sexual traits. The effects of stress on behavioral components of multi-modal traits may be especially important if the visual component requires a behavioral (e.g. postural^31,32^) adjustment in order to be seen, or if habitat choice relates directly to signalling efficiency^29,33,34^.

**Figure 1:**
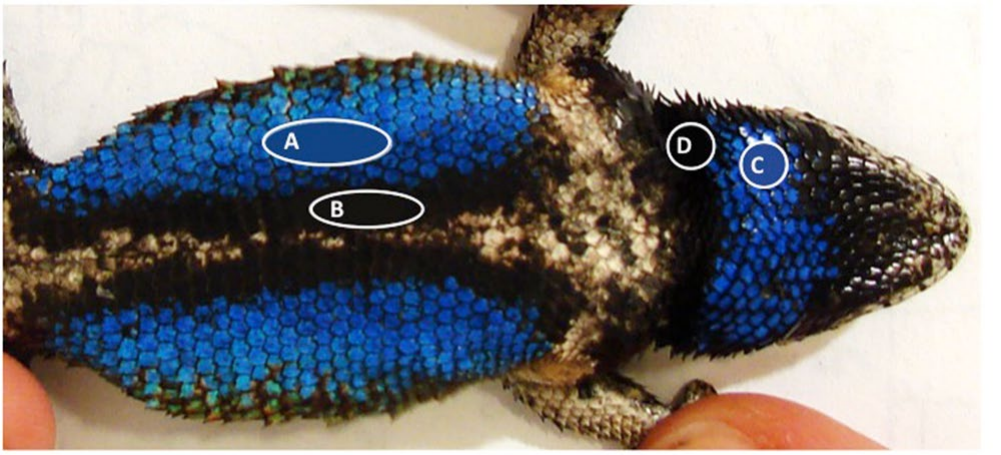
Diagram showing typical selections from the blue and black segments of *S*. *undulatus* male ventral badges (**A**,**B** – approx. 15 scales each) and the blue and black segments of gular badges (**C**,**D** – approx. 5 scales each) respectively. RGB values (intensity of the red, green and blue color components) were extracted from these selections using the Average Filter function, and the Color Dropper Tool in Adobe Photoshop (Adobe Systems Incorporated, San Jose, CA).

Here, we test the effects of physiological stress on male secondary sexual traits and behaviors relevant to their expression, in the eastern fence lizard, *Sceloporus undulatus*. Adult male eastern fence lizards have bright blue and contrasting black gular (throat) and ventral (abdominal) badges (Fig. 1) which function in sex recognition and intrasexual signalling^35^. Male badge coloration is influenced by hormones in a number of *Sceloporus* species^35–37^; for example, male secondary coloration is influenced by plasma corticosterone (corticosterone, hereafter CORT, the primary glucocorticoid in reptiles^38^) in the spiny lizard (*Sceloporus pyrocephalus*), with dull gular stripes being associated with higher CORT^37^. We therefore expect that baseline CORT will correspond with badge color, and that experimental elevations of CORT will change badge color in male fence lizards – in both cases we predict that elevated levels of CORT would be associated with paler, less vivid badges.

The ventral badges of *Sceloporus* lizards are cryptic unless displayed^35,36,39^, including through conspicuous “headbob” and “pushup” displays that expose the gular and abdominal regions^40–42^. Previous work in this genus has linked plasma CORT levels with habitat use, with hatchlings moving to higher elevations when CORT levels were experimentally elevated^43^. If adult males do the same, this could alter the visibility of their badges during display, an important factor in determining perch height selection in territorial lizards. The polygamous mating system of *Sceloporus* means that the outcome of social encounters are important for fitness^42,44^. Any changes in secondary coloration and its behavioral expression due to exposure to a stressor are therefore likely to have fitness-relevant outcomes.

We conducted three studies to examine the relationship between CORT and color and/or its use in adult male fence lizards (*Sceloporus undulatus*). First, in a Field Baseline study, we investigated the effects of glucocorticoids on male badge coloration by testing the association between baseline levels of a glucocorticoid hormone, CORT, and the color of male badge components (the blue ventral and gular segments, and the corresponding black segments). Second, we conducted a Lab Manipulation experiment, which tested causal effects of glucocorticoid elevation on male badge color using a manipulative experiment that elevated CORT to levels likely to be frequently experienced by this species. Lastly, using a Field Manipulation experiment, we investigated glucocorticoid effects on display behavior by experimentally elevating CORT levels of male lizards in the field and quantifying their display behavior and pertinent aspects of their habitat use, including time spent in refugia and perch height selection. By investigating effects on multiple aspects of this multicomponent signal, we aim to illuminate the effects of glucocorticoids elevated to ecologically relevant stress-induced levels on male secondary sexual traits in lizards.

## Methods

### Field baseline

To test for correlations between CORT and badge coloration, we captured male *S*. *undulatus* from three populations in southern Alabama (Geneva State Forest, Blakeley State Park, and Conecuh National Forest) in April and May of 2016 and 2017. Immediately upon capture we collected a blood sample from the postorbital sinus (time to bleed = 199.2 ± 13.8 seconds). Samples were kept on ice until centrifugation. Plasma was separated and frozen within 4 hrs of collection for later CORT analysis at The Pennsylvania State University. We obtained plasma samples from 46 males (N = 23 in 2015 and N = 23 in 2017). Lizards were transported in cloth bags from field sites to Solon Dixon Forestry Education Center, where they were weighed (to the nearest 0.01 g) and measured (snout-vent length, to the nearest 0.1 cm). Lizards were immediately photographed under standardized light conditions following the protocol of Langkilde & Boronow (2012). In short, the lizards’ ventral surfaces were photographed against a piece of white paper under standardized lighting: in a room illuminated only by overhead fluorescent tubes and with no windows. No auxiliary light (e.g. flash) was provided. Photographs were taken with a tripod-mounted Sony Cyber shot DSC-H5 digital camera (Sony Corporation, Tokyo, Japan). We manually set the exposure settings (shutter speed 4, ISO 100, F 2.8) and white balance to avoid inappropriate weighting of color values resulting from automatic color balance. Digital photographs do not capture light in the ultraviolet spectrum, however, like humans, lizard color photoreceptors respond best to red (long wavelength), green (medium wavelength), and blue (short wavelength) light^45^. Lizards have an additional ultraviolet wavelength-responsive color photoreceptor^45^, but *Sceloporus undulatus* badges exhibit low reflectance within UV wavelengths^46^. Lizard internal cloacal temperature was measured immediately prior to photographing using a thermocouple thermometer (Fluke Corporation, Everett, WA).

Badge color properties were extracted from photographs using Adobe Photoshop (Adobe Systems Incorporated, San Jose, CA). The white balance of each photograph was standardized, and the average color of four areas was then determined: the blue and black segments of the ventral badge, and the blue and black segments of the gular badge, using the right-side badges in all cases. An area at the centre of each segment was selected using the Elliptical Marquee Tool. The same number of scales was selected to represent each segment from each photograph: 15 scales for the ventral badge segments, and 5 scales for the gular badge segments (Fig. 1). Selecting from the same location of each badge (the centre of the badge) helped to account for variation between individuals in uniformity of color across the segment. The average color of these selections was obtained using the Average Filter function; the Color Picker Tool was then used to obtain RGB color channel values. We use RGB values instead of another commonly used metric, HSB (hue, saturation, and brightness), as HSB is based on human vision and requires image conversion^47^. We took digital photographs of a set of reflectance standards (Q13 Color Separation Guide and Gray Scale, Eastman Kodak Company, Rochester, NY, USA), fit a calibration curve, and derived a linearization equation to linearize the responses of our camera to changes in light intensity. Previous work in this species has shown that these color measurements are highly repeatable^48^.

Plasma CORT was measured via a commercially-available enzyme immunoassay (Corticosterone High Sensitivity EIA, Immunodiagnostic Systems LTD., Fountain Hills, AZ, USA) which has been previously validated for use in this species^43^. Plasma (5 uL) was brought to a final volume of 50 uL with assay buffer, and final CORT concentrations corrected for the dilution factor; all samples fell in the detectable range of the standard curve. The mean intra- and inter-assay coefficients of variation for baseline CORT were 7.8% and 13.4%. This procedure was followed for quantification of all CORT concentrations in this study.

Effects of baseline CORT on RGB values of male lizard badge segments were analysed in repeated measures MANOVAs using the statistical software JMP (Pro 13, SAS Institute Inc). The three color channels were analysed in separate models, with R/G/B values for the four badge segments (ventral blue, ventral black, gular blue, gular black) from each individual set as dependent variables to control for likely covariance in color within individuals. This model structure allows us to assess variation between individuals based on CORT levels, as well as to assess whether the badge segments change relative to each other (for example, if RGB values change in blue segments but not black, and vice versa). Initial global models for each color channel included the following independent variables: lizard internal temperature prior to photographing, SVL, body condition (residuals of log-transformed mass~SVL correlation), and baseline CORT (ng/mL, log-transformed to improve normality, hereafter logCORT). We also included year (N = 2) and site of capture (N = 6) as covariates, and an interaction term, logCORT*year, to test for different effects of baseline CORT on color between years. Non-significant terms were removed from the model (α > 0.05); logCORT was retained in all models as our term of interest. Mean values are presented as mean ± 1 standard error throughout, unless stated otherwise.

### Laboratory Manipulation

To experimentally test the effect of CORT on badge coloration, we captured 39 adult male *S*. *undulatus* from six populations across Alabama (Geneva State Forest, Conecuh National Forest), Florida (Blackwater River State Forest), Tennessee (Standing Stone State Park, Edgar Evins State Park), and Arkansas (St. Francis National Forest) in April and May of 2012. Lizards were measured and weighed upon capture (as described under Field Baseline) and transported in cloth bags to Pennsylvania State University, where they were individually housed in plastic enclosures as described in McCormick and Langkilde^49^.

Lizards were allowed to acclimate in the lab for 1.5 months (47–77 days depending on date of capture), after which they were photographed (see methods below), and randomly assigned to a treatment or control group. Lizards in the treatment group (*N* = 20) received daily application of a solution of CORT (P92%, Sigma C2505) dissolved in sesame oil (1 μg CORT/1 μL sesame oil), which resulted in the lizards receiving an average of 0.87 ug CORT per g body mass (0.50–1.25 ug/g body mass). Lizards in the control group (*N* = 19) received the sesame oil vehicle only. We applied 6 uL of either CORT solution or sesame oil only to the backs of lizards every day for 23 days. The oil and hormone were quickly absorbed due to the lipophilic nature of lizard skin^50^. A CORT-dosage in this range (0.8 μg/g body mass) results in an elevation of circulating plasma CORT concentrations approximately 30 minutes after application, which peaks at ecologically relevant stress-induced concentrations, and return to baseline levels by 90 minutes post-dosing^51^. This procedure and dosage level mimics the increase in plasma CORT after exposure to ecologically relevant stressors, such as non-lethal exposure to predatory fire ants^52,53^, heat stress (R. Telemeco, pers. comm.), chasing^43^ and restraint^54^, suggesting that elevations of CORT in this range allows us to relatively non-invasively mimic the short-term increases in CORT experienced by free-living lizards who encounter natural stressors daily. This protocol explicitly does not test the effects of pharmacologically high levels of glucocorticoids which are less likely to be frequent in natural situations^55^, and which may be better replicated using the sustained release CORT through hormone implants^56,57^. Eighteen to 21 hours after the final treatment was applied, we collected blood samples from lizards and then photographed lizards again (see Field Baseline). Plasma CORT was measured, and mean intra- and inter-assay coefficients of variation of these tests were 7.73% and 2.79%. Photography and color information extraction methods were as described under Field Baseline, with the exception that lizard temperature was measured using infrared thermometer temperature gun (Raytek MT4, Raytek Corporation, Santa Cruz, CA, U.S.A). External body temperatures obtained using this method are significantly correlated with cloacal temperatures of the same lizards obtained with a thermocouple (*P* < 0.01, D. Owen, unpublished data).

Effects of CORT treatment on RGB values of male lizard badge segments were analysed in MANOVAs using JMP. Initial global models for each color channel included the following independent variables: lizard body temperature, SVL, body condition (residuals of log-transformed mass~SVL correlation), site of capture (N = 7), and which treatment the lizard received (CORT or control). As before, non-significant terms were removed from the models (α > 0.05). There were no differences between the treatment groups in initial coloration (Red *F*_1,31_ = 1.09, *P* = 0.30; Green *F*_1,30_ = 1.41, *P* = 0.24; Blue *F*_1,30_ = 1.03, *P* = 0.32).

### Field manipulation

We conducted an experiment to test the effects of short-term increases in glucocorticoid hormones on male behavior in three populations in southern Alabama (Geneva State Forest, Blakeley State Park, and Conecuh National Forest) in April and May of 2016 and 2017. Male lizards were located in the field and given an *in situ* transdermal application of a CORT solution (4 ug CORT/1 mL of sesame oil vehicle) or a vehicle control using a fine paintbrush attached to a 6 ft fishing pole. We predict that this method transferred a mean dose volume in the order of 6.5 ± 0.67 ul, approximated by using this method to apply oil onto strips of paper which were subsequently weighed to determine volume of oil transferred (*N* = 20). The body mass of males captured over 2016 and 2017 for the baseline CORT/color study ranged from 4.2–12.7 g, with a mean of 8.8 g. Assuming a similar distribution (behavioral trials were conducted at the same sites, and therefore drew from the same populations), our dosing protocol resulted in doses ranging from 2.05–6.13 ug/g lizard body mass, with a mean dose concentration of 3.0 ug/g body mass. All doses were prepared and allocated a random number in advance so that when the numbered dose was selected haphazardly in the field, the observer was blind to treatment.

From initial sighting of the lizard, the mean time to apply the dose (“dose time”) was 83.7 seconds. After dose application, the observer moved to a distance of approximately 5 metres so as to minimize the effects of observer presence on lizard behavior. The lizard’s start position (actual location on the tree) and elevation (meters, to the nearest 0.5 m) were noted in order to calculate change in elevation, and distance moved during a trial. For a period of thirty minutes from the time of dosing, the following behaviors were recorded: number of push-ups, whether or not the lizard performed head-bobs, and how much time (min) the lizard spent in a refuge (not visible from directly above). The total distance the lizard moved and the lizard’s distance from the start position at the end of the thirty-minute period were estimated visually to the nearest 0.5 m. Elevation of the lizard (meters) was estimated to the nearest 0.5 m every five minutes, including the final elevation. Change in elevation, maximum elevation, and average elevation throughout the thirty-minute period were then calculated. Whether or not the lizard sought refuge during the trial was also recorded. The lizard was deemed to have sought refuge if it was no longer visible from directly above. Ambient temperature was measured, and cloud cover estimated visually (to the nearest 10%). Given the potential for subjectivity in estimating distance and height, only one researcher conducted trials to maximize consistency in distance and elevation estimation. Trials were discontinued if the focal lizard interacted with another lizard, as social interactions have a strong effect on behavior and CORT levels^44^. Lizards that did not have an available perch of at least 15 m within 2 m of their starting position were not used to avoid variation in maximum, average, and change in elevation due to differences in perch availability (i.e. if a lizard was on a downed log with no alternative perches nearby it would not be able to increase its perch height).

To minimize any other source of stress, lizards were not handled during these trials. Once a trial was completed, the same area (a radius of 20 m) was not resampled unless we were confident we were not resampling the same lizard, so it is likely that no male was tested twice in the same year as part of this experiment and, given low rates of recapture in these populations it is unlikely that any males were tested in both years. We conducted behavioral trials on a total of 40 males (30 from 2016, 10 from 2017): 19 were dosed with vehicle control and 21 were dosed with exogenous CORT.

The effect of treatment (CORT or control dose) on male behavior was tested in a set of linear mixed models, with the following as dependent variables: frequency of push-ups; the presence/absence of head-bobbing; total distance moved; average elevation; maximum elevation achieved; and whether or not the lizard sought refuge during the trial (0 – no, 1 – refuge sought). In all cases models include the site at which the study was conducted as a random term (N = 6). We first tested associations with year, month, dose time, cloud cover, and temperature, using single-term linear models controlling for site as a random term; where there was no significant correlation these terms were not included in final models to preserve degrees of freedom. As the presence/absence of head-bobbing was a binary variable, this model used a binomial error structure.

All protocols described adhere to the Guidelines for the Use of Animals in Research and the Institutional Guidelines of Penn State University (IACUC #35780 and # 44595), and animal collection was permitted by the respective States.

### Ethical approval

The research presented in this article adheres to the Guidelines for the Use of Animals in Research and the Institutional Guidelines of Penn State University. Approval for all procedures was granted by Penn State’s Institutional Animal Care and Use Committee (protocol numbers 35780 and 44595). Animal collection was permitted by the respective States.

### Informed consent

This article does not contain any studies with human participants performed by any of the authors.

## Results

### Field Baseline: baseline CORT and correlations with male badge color

**Figure 2:**
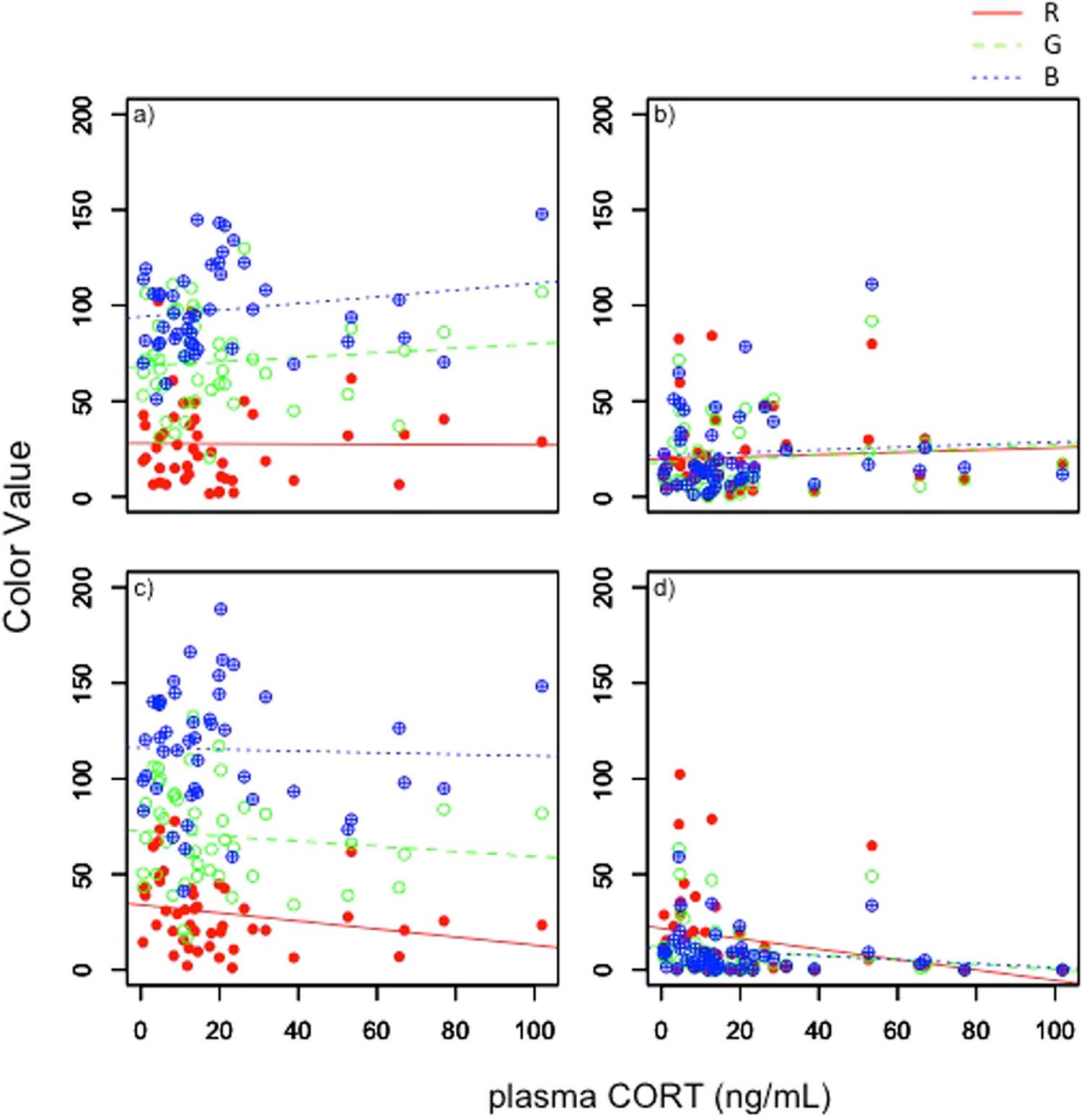
Baseline CORT in male lizards was not significantly associated (MANOVA p > 0.05) with color channel (RGB) values in ventral (**a** – blue, **b** – black) or gular badge segments (**c** – blue, **d** – black).

Baseline CORT did not predict variation in badge coloration between individuals (RGB repeated measures MANOVA: Red *F*_1,43_ = 0.19, *P* = 0.66; Green *F*_1,43_ = 1.19, *P* = 0.28; Blue *F*_1,43_ = 2.69, *P* = 0.11 Fig. 2). There were significant differences between years in all color channels across badge segments (Year: Red *F*_1,43_ = 11.84, *P* = 0.001; Green *F*_1,43_ = 6.56, *P* = 0.013; Blue *F*_1,43_ = 5.08, *P* = 0.029); badge segments were significantly lighter in 2017 (higher RGB values) than in 2016. CORT level had a significant effect on within-individual variation (i.e. between-segment) in Red color values; higher CORT levels were associated with higher Red values in chest badge segments, but reduced Red values in chin segments (*F*_3,41_ = 3.57, *P* = 0.02). Baseline CORT levels did not predict within-individual variation in Green (*F*_1,41_ = 2.73, *P* = 0.06) or Blue (*F*_1,41_ = 2.64, *P* = 0.06) values.

### Laboratory Manipulation: effects of elevated CORT on male badge color

**Figure 3:**
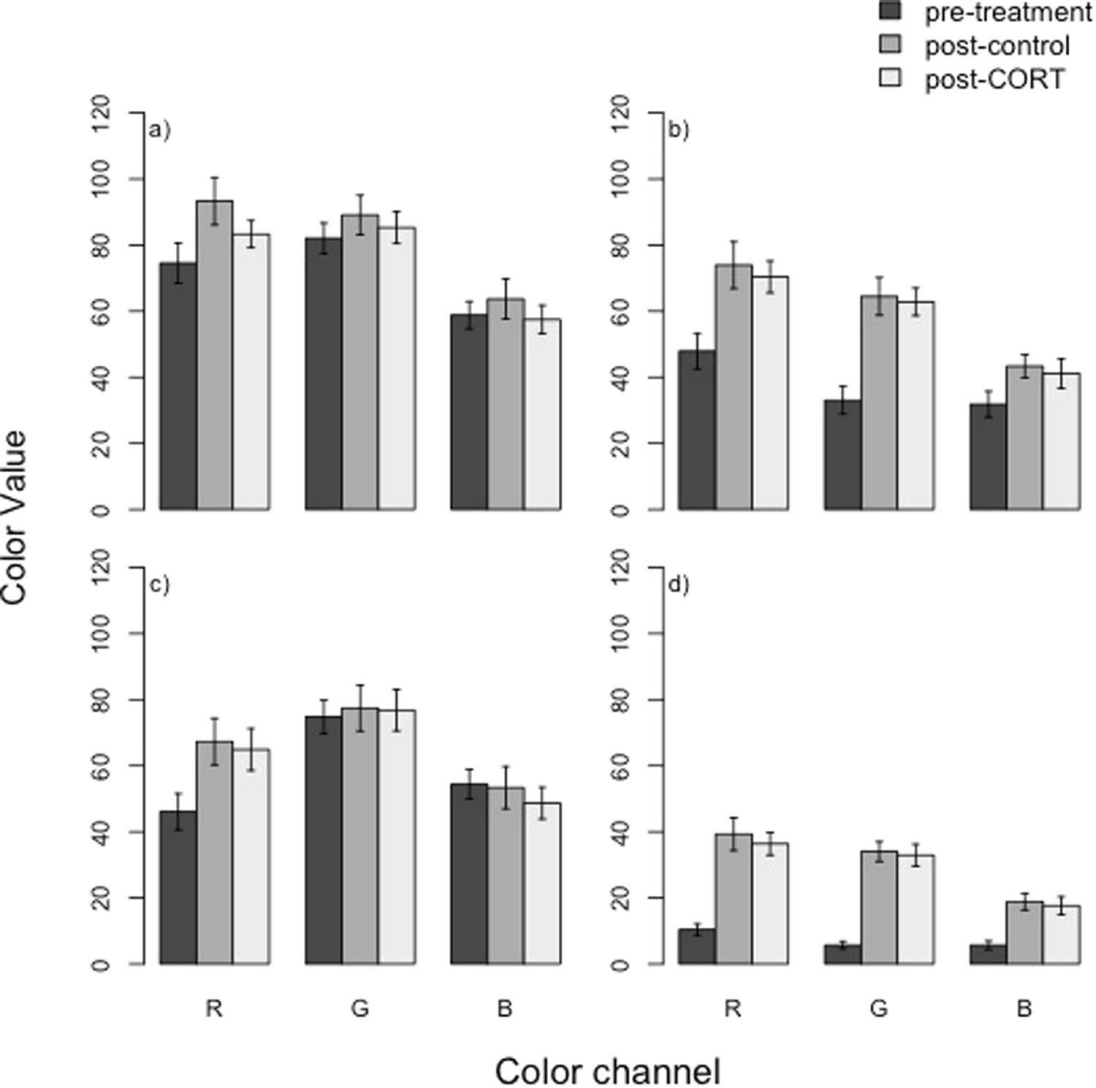
CORT and control-treated males did not significantly differ from each other in color channel (RGB) values after 23 days of treatment (MANOVA p > 0.05) in ventral badge segments (**a** – blue, **b** – black) or gular badge segments (**c** – blue, **d** – black).

CORT treatment did not result in changes in badge coloration: there were no significant differences in any color channel measured for badges of individuals that had received the CORT or control treatments (Treatment: Red *F*_1,27_ = 1.44, *P* = 0.24; Green *F*_1,27_ = 0.16, *P* = 0.69; Blue *F*_1,34_ = 0.68, *P* = 0.41; Fig. 3). In this experiment, SVL predicted color channel values, with larger individuals having lower Red and Green values, and therefore being darker overall (SVL: Red *F*_1,27_ = 8.21, *P* = 0.008; Green *F*_1,27_ = 9.76, *P* = 0.004). Blue values were not affected by SVL (*F*_1,27_ = 3.28, *P* = 0.08). There was also significant variation between sites of initial capture in Red and Green values (Site: Red *F*_6,27_ = 3.58, *P* = 0.01; Green *F*_6,27_ = 2.90, *P* = 0.03), but not in Blue values (*F*_1,27_ = 1.41, *P* = 0.25).

### Field Manipulation: effects of elevated CORT on male signalling behavior

CORT treatment did not affect the number of push-ups performed by male lizards (*T*_*1*,*39*_ = −0.58, *P* = 0.58), the likelihood of males displaying head-bobbing behavior (*Z*_*1*,*39*_ = 0.29, *P* = 0.77), or the total distance (m) male lizards moved during the trial period (*T*_*1*,*38*_ = 0.037, *P* = 0.97). The higher the percentage of cloud cover during a trial, the less the male lizards moved overall (*T*_*1*,*38*_ = −3.61, *P* < 0.001). Thirteen males sought refuge during trials; there was no significant effect of treatment on this behavior (5 CORT-treated and 8 control lizards sought refuge; *Z*_*1*,*39*_ = −0.73, *P* = 0.46).

**Figure 4:**
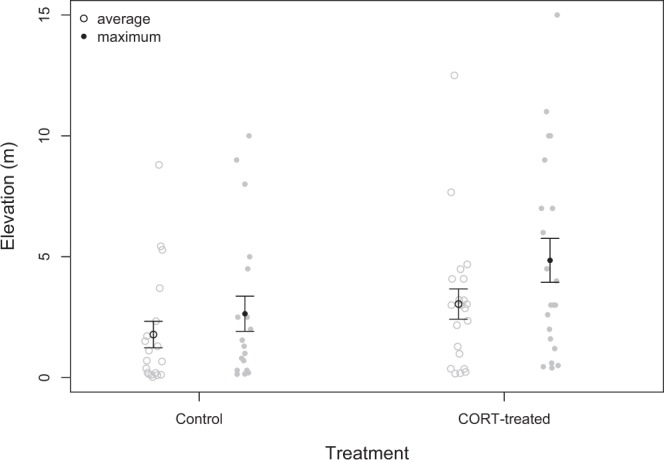
Male lizards treated with CORT in the field perched higher on average during trials than controls (*P* = 0.06) and trended to have a greater maximum height during the trial period. Individual data points are shown in grey. Average elevation values in both treatment groups are shown as open circles, and maximum elevation values in both treatment groups are shown as solid circles. Mean ± 1 SE for the control and CORT-treated groups are overlaid in black.

Although all trials were started *in situ* and there was thus variation between male start elevation, start elevation did not differ between treatment groups (Kruskal Wallis X^2^_1_ = 0.31, *P* = 0.58); differences in average and maximum height therefore represent movement to higher elevations during the trial itself. Treatment had some influence on the height at which male lizards chose to perch (Fig. 4): there was a trend for CORT-treated lizards to maintain a higher average height during trials; however, this result did not achieve statistical significance (*T*_*1*,*39*_ = 1.84, *P* = 0.06, control mean 1.78 m ± 0.55, CORT-treatment mean 3.0 m ± 0.6). The maximum height attained during trials was greater for the CORT-treated than control males (*T*_*1*,*39*_ = 2.00, *P* = 0.04, control mean 2.6 m ± 0.7, CORT-treatment mean 4.9 m ± 0.9), though this effect is driven largely by one male that achieved a maximum height of 15 m in the CORT-treated group (there was no significant effect of treatment with this male excluded: *T*_*1*,*38*_ = 1.59, *P* = 0.10).

## Discussion

Exposure to environmental stressors has long been thought likely to influence aspects of sexual signals and traits through direct or indirect effects of glucocorticoid hormones such as corticosterone (CORT)^9^. Studies have focused largely on long-term stress or glucocorticoid exposure which may not represent environmental stimuli at ecologically relevant levels in wild-living animals. Here, we explicitly test the effects of glucocorticoid elevation on sexual signalling traits at ecologically relevant levels and timescales. Short-term stimuli have the potential to alter iridophore- and melanin-based coloration^58^, such as that in *Sceloporus*^39^. However, we show using a combination of baseline CORT measurements and CORT manipulations in the field and laboratory that CORT did not influence gular or ventral badge coloration, or signalling behavior, in wild-caught eastern fence lizards. These traits may be maintained in the face of stressors due to their importance in determining male fence lizard fitness through acquiring and maintaining territories^42,44^; alternatively, these traits may not be strongly condition-dependent, or simply not affected by short-term changes in CORT. However, there was some indication that male lizards altered their perch height selection, which may have consequences for signal perception. It is difficult to assess whether our results represent a common lack of a link between ecologically-relevant levels of physiological stress and coloration/signalling behavior, or are more anomalous given a likely publication bias toward significant results in ecology and evolution^59^.

There is cross-taxa evidence for effects on secondary sexual traits of environmental stressor-exposure, and/or specific components of the physiological stress response^10–12,20–23,25^. In lizards, (carotenoid-based) coloration can be influenced by glucocorticoid concentrations^20,21^. However, the evidence for glucocorticoid effects on color traits in sceloporine lizards is mixed. Blue gular stripe brightness correlated negatively with plasma CORT in male spiny lizards (*Sceloporus pyrocephalus*)^37^, but no correlation existed between CORT and gular/ventral melanin in the western fence lizard (*Sceloporus occidentalis*)^60^. It is possible that the characteristic badges in these lizards are robust to environmental and physiological change; for example, male *Sceloporus* badge characteristics display a lack of change according to seasonal change^61^ and fluctuations in testosterone^37^, potentially suggesting a lack of condition-dependence in this trait. Although sexual signalling traits are often assumed to be condition-dependent, in line with the Handicap Principle^62^, there is surprisingly little empirical support for this^63^. Badge coloration in *S*. *undulatus* alters according to temperature^64^, suggesting the ability of this trait to display some lability in the face of short-term environmental variation. The blue coloration in these badges is formed by structural plates which reflect the light. These may be largely permanent once formed, though the distance between these structural plates is temperature-sensitive, altering the reflected color (see^64^). We showed no link between baseline CORT levels and badge coloration (in terms of RGB values) despite considerable variation in CORT levels. This was supported up by our experimental manipulation, which also found no effects of elevated CORT levels.

In addition to a lack of influence of CORT on secondary sexual trait coloration (baseline and manipulated levels), we found no effect of short-term CORT elevation on signalling and related territorial behaviors in wild male fence lizards. Specifically, *in situ* CORT treatment did not alter the rate of pushup and head-bob displays, how far lizards moved, and whether they sought refuge. We predicted that signalling behavior may change, given that numerous studies have found behavioral effects of glucocorticoids: for example, common wall lizards (*Lacerta vivipara*) show increased locomotor activity when CORT is increased^50,65^. Elevated plasma CORT has also been shown to increase antipredator behaviors in lizards (including *Sceloporus*), such as hiding or fleeing^43,66,67^. Nevertheless, these results are in line with a meta-analysis that found no correlation between physiological correlates of stress and the expression of sexual signals across 26 vertebrate species^68^. Despite no signficant trends for “stress” effects on vocalisation, morphological, or color traits, there was a significant opposite-sex preference for low-stress individuals^68^. Therefore, although male fence lizards in our study did not show differences in expression of sexual traits, there may still be a cost of higher glucocorticoid levels in terms of female preference that we were not able to quantify. Further, the maintenance of signalling and signalling behaviors in the face of higher glucocorticoids may represent a trade-off between display and survival with downstream costs that were beyond the scope of this study^69,70^. For example, the trade-off between signalling investment and survival in field crickets^71^.

Although we found no effects of CORT on secondary sexual coloration or signalling or signalling-relevant behavior, there was a trend for CORT-treated male lizards to move to higher perching elevations, with a mean maximum perch height almost double that of the males that received a control dose. This ~2.5 m difference in perch height has the potential to affect the perception of the secondary sexual signals by conspecifics be removing the male from their line of sight. Although we did not test aspects of signal perception by either conspecific females or rival males, we suggest that perception, and how habitat use might influence perception, is often neglected in the study of sexual signalling, particularly how it is affected by physiological change. Our results emphasize the importance of considering the effects of physiological traits such as glucocorticoids on secondary sexual traits in a holistic way that takes into account species ecology as well as morphology and behavior.

## Data Availability

All data are available at 10.18113/D3Q67N.
